# Mitochondrial Iron Overload-Mediated Inhibition of Nrf2-HO-1/GPX4 Assisted ALI-Induced Nephrotoxicity

**DOI:** 10.3389/fphar.2020.624529

**Published:** 2021-01-21

**Authors:** Hui-Fang Deng, Lan-Xin Yue, Ning-Ning Wang, Yong-Qiang Zhou, Wei Zhou, Xian Liu, Yu-Hao Ni, Cong-Shu Huang, Li-Zhen Qiu, Hong Liu, Hong-Ling Tan, Xiang-Lin Tang, Yu-Guang Wang, Zeng-Chun Ma, Yue Gao

**Affiliations:** ^1^ Department of Pharmaceutical Sciences, Beijing Institute of Radiation Medicine, Beijing, China; ^2^ Tianjin University of Traditional Chinese Medicine, Tianjin, China; ^3^ School of Traditional Chinese Medicine, Guangdong Pharmaceutical University, Guangzhou, China

**Keywords:** aristolactam I, nephrotoxicity, ferroptosis, mitochondrial iron overload, Nrf2-HO-1/GPX4

## Abstract

Aristolactam I (ALI) is an active component derived from some Traditional Chinese medicines (TCMs), and also the important metabolite of aristolochic acid. Long-term administration of medicine-containing ALI was reported to be related to aristolochic acid nephropathy (AAN), which was attributed to ALI-induced nephrotoxicity. However, the toxic mechanism of action involved is still unclear. Recently, pathogenic ferroptosis mediated lipid peroxidation was demonstrated to cause kidney injury. Therefore, this study explored the role of ferroptosis induced by mitochondrial iron overload in ALI-induced nephrotoxicity, aiming to identify the possible toxic mechanism of ALI-induced chronic nephropathy. Our results showed that ALI inhibited HK-2 cell activity in a dose-dependent manner and significantly suppressed glutathione (GSH) levels, accompanying by significant increases in intracellular 4-hydroxynonenal (4-HNE) and intracellular iron ions. Moreover, the ALI-mediated cytotoxicity could be reversed by deferoxamine mesylate (DFO). Compared with other inhibitors, Ferrostatin-1 (Fer-1), a ferroptosis inhibitor, obviously alleviated ALI-induced cytotoxicity. Furthermore, we have shown that ALI could remarkably increase the levels of superoxide anion and ferrous ions in mitochondria, and induce mitochondrial damage and condensed mitochondrial membrane density, the morphological characteristics of ferroptosis, all of which could be reversed by DFO. Interestingly, ALI dose-dependently inhibited these protein contents of nuclear factor erythroid 2-related factor 2 (Nrf2), heme oxygenase-1 (HO-1), and glutathione peroxidase 4 (GPX4), which could be partly rescued by Tin-protoporphyrin IX (SnPP) and mitoTEMPO co-treatment. In conclusion, our results demonstrated that mitochondrial iron overload-mediated antioxidant system inhibition would assist ALI-induced ferroptosis in renal tubular epithelial cells, and Nrf2-HO-1/GPX4 antioxidative system could be an important intervention target to prevent medicine containing ALI-induced nephropathy.

## Introduction

Traditional Chinese medicine (TCM) has excellent advantages in prevention and treatment of complex and chronic diseases, for instance, chronic kidney disease (CKD) (Wang K-X et al., 2020). For people who suffered from CKD, TCM has been widely used as an effective alternative therapy in China and many Asian countries. In the clinical practice, the classical Chinese herbal formulas, such as Zhenwu Decoction (La et al., 2018) and Dahuang Fuzi Decoction (Tu et al., 2014), have been widely applicated in the treatment of CKD and exerted good efficacy in alleviating symptoms, improving renal function, preventing and treating complications, and delaying the development of renal failure. However, some herbal medicines containing toxic compounds have been confirmed to exist in these formulas, for instance, Xixin, a kind of TCM which contains aristolochic acid analogs (AAAs) and is known for its nephrotoxicity (Gökmen and Lord, 2012; Michl et al., 2017).

Aristolactam I (ALI) is one of the potential nephrotoxic AAAs in Asarum species, also the major reductive metabolite of aristolochic acid I (AAI), which is considered to be the most important constituent causing aristolochic acid nephropathy (AAN) (Bastek et al., 2019). Interestingly, it had been reported that the nephrocytotoxicity of ALI might be stronger than that of AAI *in vivo* (Li et al., 2016). Li et al. found that IC_50_ of ALI for HK-2, the human proximal tubular epithelial cell line, was 25 μM, and was less than that of aristolochic acid I (45 μM) (Li et al., 2010), which mean that long-term use of TCM containing ALI would produce potential risk of kidney injury. A number of studies had assessed the toxicity and carcinogenicity of AAI, but little is known about the nephrotoxicity of ALI and related toxic mechanisms. Previous studies have demonstrated that the most implicated mechanisms of AA-induced nephrotoxicity include apoptosis, oxidative stress, and inflammation (Gökmen and Lord, 2012), all of which might be involved in ALI-induced nephrotoxicity.

Ferroptosis, a new form of regulated cell death identified in recent years, is characterized by the iron-dependent overwhelming accumulation of lipid hydroperoxides (Dixon et al., 2012; Stockwell et al., 2017). It is morphologically, biochemically and genetically distinct from other types of known regulated cell death, such as apoptosis, autophagy, and necrosis, pyroptosis. Ferroptosis is mainly characterized by increased ferrous ion, lipid oxidation, as well as increased mitochondrial membrane density (Dixon et al., 2012). Numerous studies have demonstrated ferroptosis played a critical role in brain, kidney, and heart pathology (Wang et al., 2017) and recently has been verified to be implicated in diverse kidney diseases, such as acute kidney injury, polycystic kidney disease and renal cell carcinoma (Tang and Xiao, 2020). However, there is no studies having reported the role of ferroptosis in ALI-induced nephrotoxicity for now.

In general, the most recognized trigger of ferroptosis is the intracellular iron imbalance which is always induced by oxidative reaction. Mitochondrion is considered to play a pivotal role in the execution of ferroptosis, for it is the major organelle of iron metabolism and ROS generation (Wang H et al., 2020). The latest research demonstrated that increased intracellular iron uptake and accumulation could contribute to excessive cytoplasmic iron influx into mitochondria to participate in the synthesis of Fe-S clusters (Wang H et al., 2020), implying that mitochondrial iron overload could accelerate the process of ferroptosis. Nuclear factor erythroid 2-related factor 2 (Nrf2) and heme oxygenase 1 (HO-1) are well documented as critical antioxidative enzymes, both of which could be inducible and participate in the metabolism of intracellular iron, the synthesis of glutathione peroxidase 4 (GPX4), and the regulation of intracellular iron concentration, to protect from oxidative damage induced by ferroptosis (Song and Long, 2020). However, metabolomics analysis revealed that Nrf2 dysfunction was found in AAN (Zhao et al., 2015), indicating a possible inhibition of Nrf2-HO1 signaling pathway in ALI-induced kidney injury.

In this study, we assumed that ALI induced ferroptosis by increasing intracellular Fe^2+^ accumulation and lipid hydroperoxide, meanwhile promoted the overload of mitochondrial labile iron, which inevitably led to the generation of excessive superoxide anion and in turn inhibited Nrf2-HO-1/GPX4 antioxidative enzyme system and enhanced labile iron release to assist ALI-induced ferroptosis. This vicious cycle would be the key toxic mechanism underlying ALI-caused chronic nephropathy.

## Methods and Materials

### Cell Culture and Treatments

Human proximal tubular epithelial cell line (HK-2) was obtained from the National Infrastructure of Cell Line Resource (Beijing, China). HK-2 cells were cultured in DMEM (Thermo Fisher Scientific, Waltham, MA, United States) with 10% fetal bovine serum and penicillin-streptomycin (0.1 kU·ml^−1^-0.1 mg ml^−1^, FG101–01, Transgene, China), and placed in a humidified incubator at 37°C containing 5% CO_2_. Aristolactam I (ALI) was purchased from Solaribo (SA9650, Solaribo life science). Z-VAD-FMK (Z-VAD) and Necrostatin-1 (Nec-1) were obtained from Beyotime Biotechnology (C1202 and SC4359, Shanghai, China). 3-Methylamine (3-MA) and Tin-protoporphyrin IX (SnPP) were purchased from MedChemExpress (HY-19312 and HY-101194, USA). Deferoxamine mesylate (DFO) was from Solarbio Life Science (D5760, China). (2-(2,2,6,6-Tetramethylpiperidin-1-oxyl-4-ylamino)-2-oxoethyl) triphenylphosphonium chloride (mitoTEMPO or MT) (SML0737, ≥98% (HPLC)) and Ferrostatin-1 (Fer-1) (SML0583, ≥95% (HPLC)) were acquired from Sigma-Aldrich (China).

### Cell Viability Assay

Cell viability was detected using Cell Counting kit-8 assay (CCK-8, Dojindo, Japan) according to the manufacturer’s instruction. 6,000 cells were seeded in a 96-well plate per well. After growing for 24 h, cells were treated with gradient concentrations of ALI (0–50 μM), cell death inhibitors—Fer-1 (2 μM, pretreated for 2 h), Nec-1 (1 μM, cotreated), Z-VAD (2 μM, cotreated), 3-MA (5 mM, cotreated) and DFO (50 μM, pretreated for 2 h) and their combination with ALI for 24 h. After treatment, cells were washed with PBS buffer gently and incubated with DMEM medium containing 10% CCK-8 solution for 2 h in an incubator with 5% CO_2_ at 37°C. Then, the absorbance of each well was determined with a microplate reader at 450 nm (Multiskan MK3, Thermo Fisher Scientific, United States). The cell viability of the treatment group was expressed as a percentage relative to that of the control group.

### Determination of Glutathione (GSH)

Monobromobimane (MBB, MedChemExpress, United States), an essentially nonfluorescent substance, but convertible to fluorescent products when reacts with small thiol, which GSH owns, was used to detect GSH. HK-2 cells (5 × 10^4^ cells/ml) were seeded in 35-mm plates and grew for 24 h. Then, cells were exposed to 3.125, 6.25, 12.5, 25, 50 μM of ALI, pretreated with 50 μM DFO for 2 h at first or not for 24 h. Controls were cultured with complete medium. After treatment, medium DMEM was removed and cells were washed three times with HBSS (Thermo Fisher Scientific, Waltham, MA, United States). Thereafter, 2 ml of MBB solution (100 μM) was added. After 15 min in the dark at 37°C, the fluorescence intensity of the MBB-GSH conjugate was recorded by an inverted fluorescence microscope (Ti2, Nikon, Japan). Processing of the pictures was achieved with ImageJ.

### Transmission Electron Microscopy

After ALI treatment with or without DFO, cells were collected and centrifuged at 1,000 rpm for 10 min, supernatant discarded. Then, the pre-cooled 2.5% glutaraldehyde was added to fix overnight at 4°C. The next day, cells were washed with ice-cold PBS, and fixed with 1% osmium tetroxide at 4°C for 30 min. At room temperature, the cell samples were dehydrated using gradient ethanol. Sequentially, the sample was embedded with epoxy resin and cut into 1 μm ultrathin sections. Having been processed, cell samples were subject to a transmission electron microscope (H-7650, Hitachi, Tokyo, Japan) to observe the ultrastructure of cells, mainly focusing on the mitochondria.

### Intracellular Iron Determination

The extranuclear iron concentration was detected by the iron ion colorimetric detection kit (E1042, Applygen Technologies, China). The HK-2 cells were seeded in 6-well plates at a density of 1 × 10^5^/ml. Then the cells were treated with ALI for 24 h. After treatment, the cells were collected and lyzed with RIPA buffer (C1053+, Applygen Technologies, China). According to the manufacture’s instruction, a standard stock solution was prepared immediately using the dilute solution. Mixture A was prepared by mixing the buffer solution with 4.5% potassium permanganate solution at the ratio of 1:1. Next, thoroughly mix the sample with the detection working solution, and incubate at 60°C for 1 h. At the end of the incubation, the iron ion detection reagent was added and incubated at room temperature for 30 min 200 μL of the final solution was added to a 96-well plate per well, and the concentration of iron ions in the cells was detected at 550 nm using a microplate reader (Multiskan MK3, Thermo Fisher Scientific, United States).

### Western Blot Analysis

The concentrations of whole-cell proteins were qualified by a bicinchoninic acid (BCA) protein assay kit (Applygen Technologies, Beijing, China). The separation of proteins was completed by sodium dodecyl sulfate-polyacrylamide gel electrophoresis (SDS-PAGE). Proteins were then transferred to PVDF membranes (IPVH00010, Millipore, Germany), blocked in blocking buffer for 2 h. After that, the membranes were incubated with the following primary antibodies: Rabbit Anti-Nrf2 (phospho S40) Monoclonal antibody (ab76026, Abcam), 4-Hydroxynonenal Mouse Monoclonal antibody (MAB3249, R&D systems), HO-1/HMOX1 Rabbit Polyclonal antibody (10701-1-AP, proteintech), GPX4 Rabbit Polyclonal antibody (14432-1-AP, proteintech) and beta Actin Mouse Monoclonal antibody (66,009-1-lg, proteintech). Then, the membranes were washed and incubated with secondary antibodies (Goat Anti-Rabbit IgG H&L (HRP) (ab6721, Abcam) and Goat Anti-Mouse IgG H&L (HRP) (ab6789, Abcam)). Following the manufacture’s instruction, the expression of proteins was detected by an enhanced chemiluminescence system (Millipore, Germany). ImageJ software was used for each band’s intensity detection.

### Detection of Mitochondrial Ferrous Iron (Fe^2+^) Using Mito-FerroGreen

HK-2 cells were seeded on 20 mm glass-bottomed culture dishes at a density of 2.5 × 10^4^/ml. After 24 h, cells were subject to different groups, control, ALI (25 μM), ALI + DFO (50 μM, pretreated for 2 h), ALI + SnPP (20 μM, cotreated), ALI + MT (0.5 μM, cotreated) for 24 h, then the medium was discarded and cells were washed with HBSS for three times. Then, 5 μM Mito-FerroGreen (Dojindo, Japan) working fluid was added to the cells. The system was then incubated for 30 min at 37°C in a 5% CO_2_ incubator to combine probes with mitochondrial Fe^2+^. After discarding the supernatant, 10 mM deferoxamine mesylate salt prepared with HBSS was added to the cells. After another 30 min’ incubation under the same condition, the liquid was discarded and the cells were washed with HBSS three times. At last, the cells were observed by a confocal fluorescence microscope (Leica TCS-SP2 confocal microscope) at (Ex/Em) 488 nm/510–550 nm.

### Mitochondrial Superoxide Determination

HK-2 cells were seeded and cultured as described for detection of mitochondrial Fe^2+^, and control, ALI, ALI + DFO, ALI + MT groups were set. Mitochondrial ROS (mtROS) was detected by MitoSOX™ Red mitochondrial superoxide indicator (M36008, Thermo Fisher Scientific, United States). According to the manufacture’s instruction, cells were loaded by adding the 5 μM MitoSOX™ reagent working solution prepared and then incubated for 20 min at 37°C, protected from light. After that, cells were washed and stained with PBS for imaging. Pictures were captured by a confocal microscope above at (Ex/Em) 510/580 nm. The level of mtROS was expressed as fluorescence intensity.

### Statistical Analysis

All results were presented as mean ± standard deviation (SD) and generated from at least three independent experiments. One-way ANOVA followed by LSD post hoc test for multiple comparisons was performed for statistical analysis. Only at *p* < 0.05 was considered significant.

## Results

### ALI-Induced Cytotoxicity on HK-2 Cells Was Associated With Ferroptosis

**FIGURE 1:**
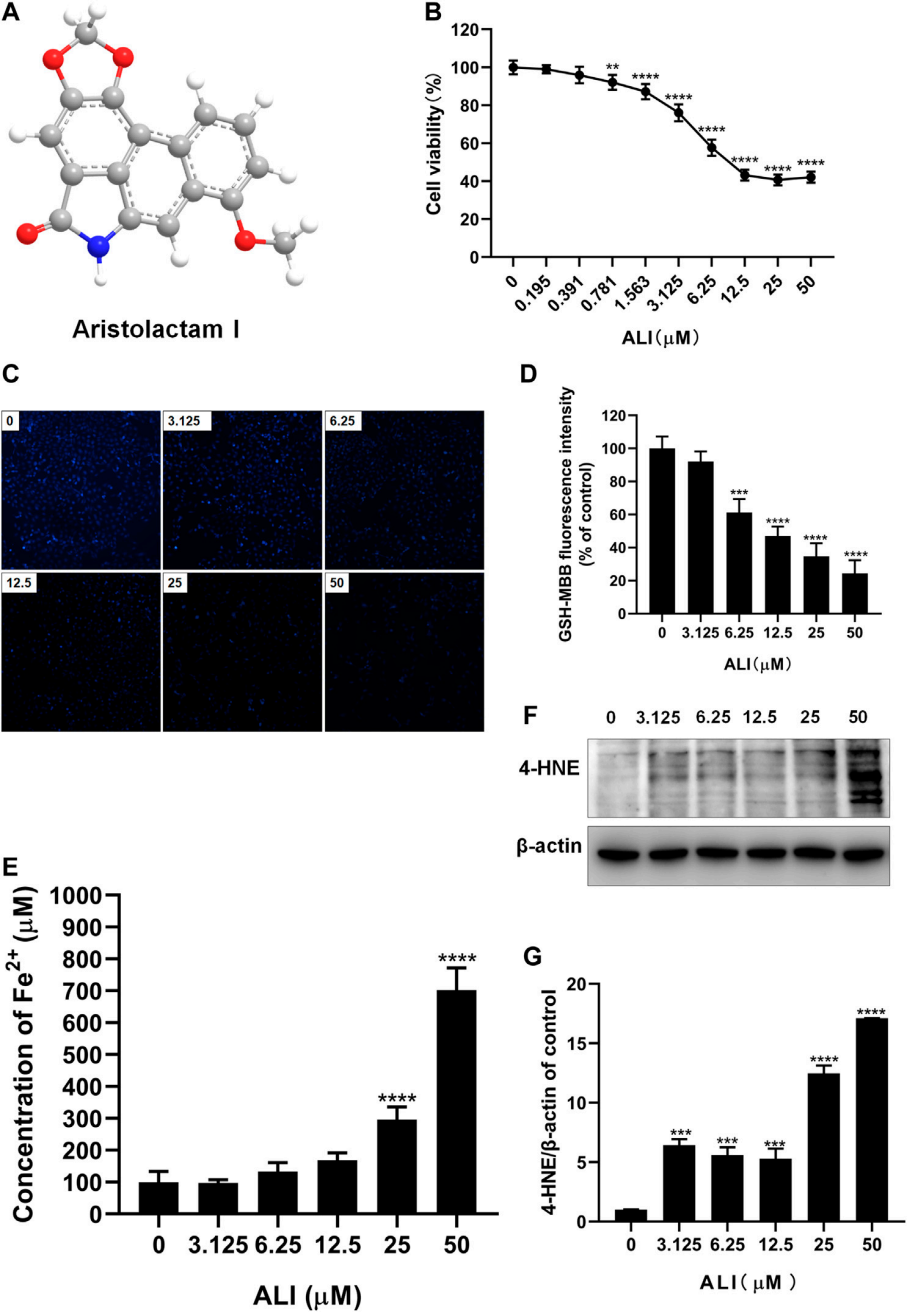
ALI-induced cytotoxicity on HK-2 cells was associated with ferroptosis. **(A)** Chemical structure of ALI. **(B)** Cell viability of HK-2 cells after 24 h ALI treatment was detected using CCK-8 assay. **(C)**, **(D)** Intracellular GSH content in HK-2 (X200). **(E)** Intracellular Fe^2+^ levels in HK-2 cells treated with ALI. **(F)**, **(G)** The protein levels of 4-HNE were measured by Western blot. ^*^ *p* < 0.05, ^**^ *p* < 0.01, ^***^ *p* < 0.001, ^****^ *p* < 0.0001 vs. the control group.

To determine the cytotoxicity of ALI on renal tubular epithelial cells, HK-2 cells were exposed to various concentrations of ALI (0–50 μM, Figure 1A) for 24 h. The CCK-8 result showed that the toxicity of ALI to HK-2 cells was dose-dependent (Figure 1B). As shown in Figures 1C and 1D, the fluorescence intensity of MBB-GSH conjugate also dose-dependently decreased, suggesting the decreases of GSH content in cells. In addition, the ferrous ions obviously accumulated in cells along with ALI concentration increased (Figure 1E), indicating that ALI could induce intracellular iron overload. Additionally, Western blot results showed that the expression of lipid peroxidation product, 4-HNE, a classic indicator of ferroptosis-induced lipid hydroperoxides, gradually enhanced as the increase of ALI concentration (Figures 1F and 1G). All results indicated that ferroptosis might participate in ALI-induced cytotoxicity on HK-2 cells.

### ALI-Induced Cytotoxicity Could Be Alleviated by Iron Chelator DFO

**FIGURE 2:**
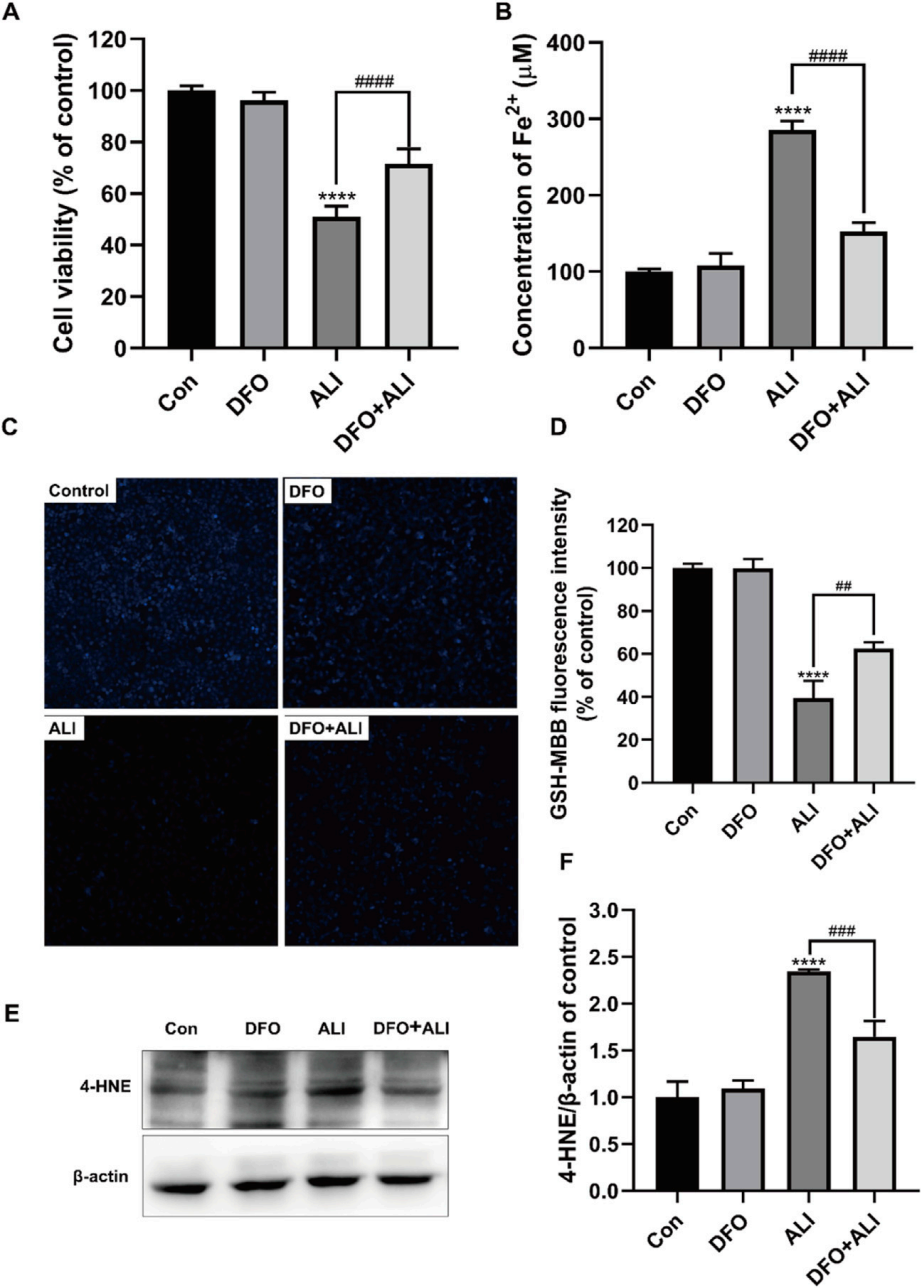
ALI-induced cytotoxicity could be alleviated by iron chelator DFO. **(A)** Cell viability of HK-2 cells was detected using CCK-8 assay. **(B)** Intracellular Fe^2+^ levels in HK-2 cells treated by ALI and DFO. **(C,D)** Intracellular GSH content in HK-2 (X200). **(E,F)** The protein levels of 4-HNE were measured by Western blot. *p < 0.05, **p < 0.01, ***p < 0.001, ****p < 0.0001 vs. the control group; *^#^p* < 0.05, *^##^p* < 0.01, *^###^p* < 0.001, *^####^p* < 0.0001 vs. the ALI group.

To further assure the role of ferroptosis in ALI-induced HK-2 cell death, DFO, a well-known agent that can inhibit ferroptosis by chelating ferrous ions, was used to prevent ALI-induced ferroptosis. As shown in Figure 2A, pretreatment with DFO (50 μM) for 2 h significantly improved the cell viability, which was decreased by ALI treatment. DFO efficiently ameliorated the overload of Fe^2+^ (Figure 2B) and the decreased GSH content caused by ALI (Figures 2C and 2D). Moreover, compared to the ALI group, Western blot results showed that the level of 4-HNE was significantly reduced after 2 h of DFO pretreatment (Figures 2E and 2F). These results demonstrated that ferroptotic cell death caused by ALI could be attenuated by DFO, suggesting that ALI-induced cell death was associated with ferroptosis.

### Ferroptosis Was the Predominant Pattern of HK-2 Cell Death Induced by ALI

**FIGURE 3:**
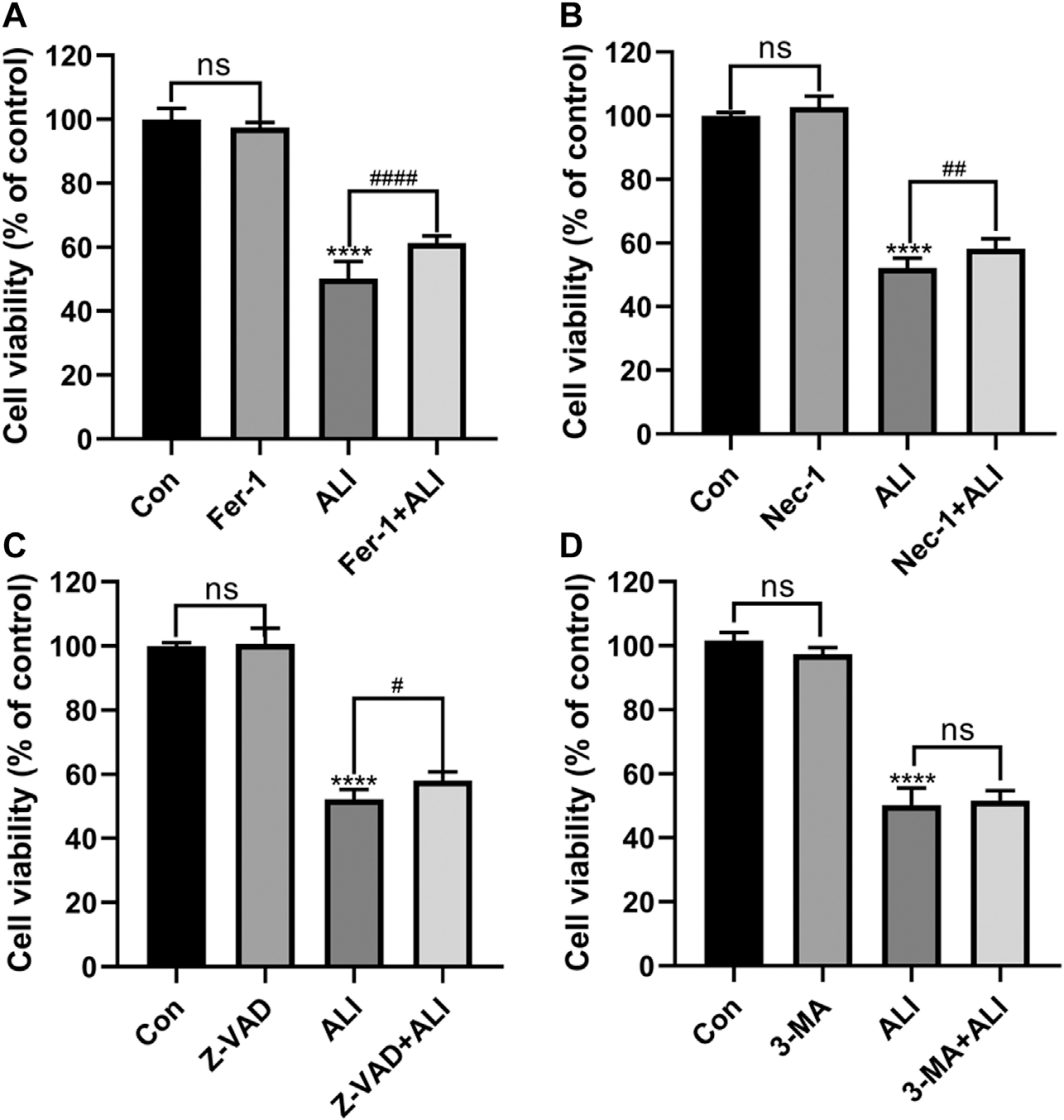
Ferroptosis was the predominant pattern of HK-2 cell death induced by ALI. **(A)** HK-2 cells were pretreated with 2 μM Ferrostatin-1 for 2 h and then exposed to 25 μM of ALI or not for 24 h. Cell viability of HK-2 cells was detected using CCK-8 assay. **(B)** Use CCK-8 assay to detect the cell viability of HK-2 cells after 24 h of co-treatment with ALI and Necrostatin-1. **(C)** CCK-8 assay was used to detect the effect of ALI and Z-VAD on HK-2 cell viability. **(D)** CCK-8 assay was used to detect the effect of ALI and 3-MA on the cell viability of HK-2 cells. ns, no significant; ^*^ *p* < 0.05, ^**^ *p* < 0.01, ^***^ *p* < 0.001, ^****^ *p* < 0.0001 vs. the control group; ^#^ *p* < 0.05, ^##^ *p* < 0.01, ^###^ *p* < 0.001 vs. the ALI group.

Next, different inhibitors of cell death, Fer-1, 3-MA, and Z-VAD, Nec-1, which specifically inhibit ferroptosis, autophagy, and apoptosis, necrosis, respectively, were used to verify the predominant cell death pattern induced by ALI. As shown in Figure 3, all the inhibitors alone had no significant effect on cell survival. Fer-1, Z-VAD, and Nec-1 could significantly reverse the decrease of cell viability caused by ALI, among which pretreatment of Fer-1 significantly improved the cell viability by 11% (Figure 3A) but Z-VAD and Nec-1 both only elevated the cell viability by about 6% (Figures 3B and 3C). And 3-MA exerted no effects on HK-2 cell viability (Figure 3D), indicating that autophagy might not be involved in cell death caused by ALI. These results indicated that ferroptosis was the main pattern of HK-2 cell death induced by ALI.

### Mitochondrial Iron Overload and mtROS Were Involved in ALI-Induced Cytotoxicity

**FIGURE 4:**
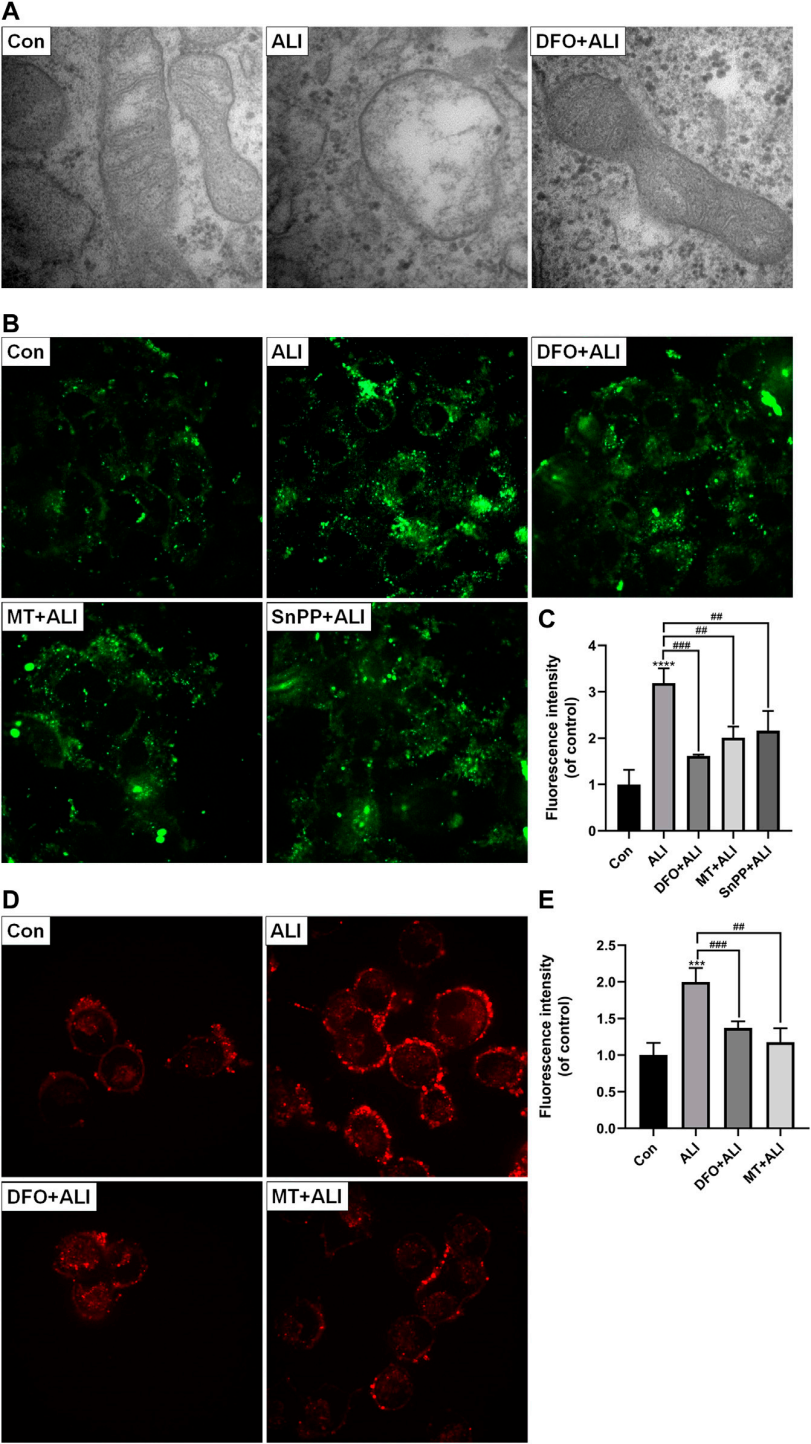
Mitochondrial iron overload and mtROS were involved in ALI-induced cytotoxicity. **(A)** Electron micrograph images of HK-2 cells treated by ALI pretreated with or without DFO to display mitochondrial microstructure. **(B)**, **(C)** Mitochondrial Fe^2+^ levels in HK-2 cells were detected using Mito-FerroGreen probe. **(D)**, **(E)** The mitochondrial superoxide anion levels in HK-2 cells were assessed using MitoSOX. ^*^ *p* < 0.05, ^**^ *p* < 0.01, ^***^ *p* < 0.001, ^****^ *p* < 0.0001 vs. the control group; ^#^ *p* < 0.05, ^##^ *p* < 0.01, ^###^ *p* < 0.001 vs. the ALI group.

Mitochondria, as the core of redox homeostasis, play a critical role in ferroptosis. As shown in Figure 4A, the morphology of mitochondria in the control group was normal, characterizing by complete membrane structure and normal mitochondrial cristae. However, in the ALI group, smaller and rounder mitochondria appeared accompanying with denser membrane and cavitation, which was a typical feature of ferroptosis. But with DFO pretreatment, the mitochondrial damage was obviously reversed. The results of Mito-FerroGreen staining (Figures 4B and 4C) showed that the fluorescence intensity of the ALI group was significantly higher than that of the control group, indicating that ALI induced a significant increase in free iron ions in mitochondria. At the same time, levels of mtROS in HK-2 cells were elevated significantly by ALI (Figures 4D and 4E). To figure out the relationship between mitochondrial Fe^2+^ overload and mitochondrial ROS increase, we performed the co-treatment of iron chelator DFO or mtROS scavenger MT with ALI. As Figures 4C and 4E showed, the Mito-FerroGreen and MitoSOX fluorescence intensity of the DFO + ALI group and MT + ALI group were lower than that of the ALI group. These results indicated that mtROS generation was involved in the balance of iron ion homeostasis in mitochondria, and the renal cytotoxicity of ALI might be related to mitochondrial iron overload-induced by mitochondrial oxidative stress.

### Ferroptosis Induced by ALI Was Related to the Inhibition of Nrf2-HO-1/GPX4 Pathway

**FIGURE 5:**
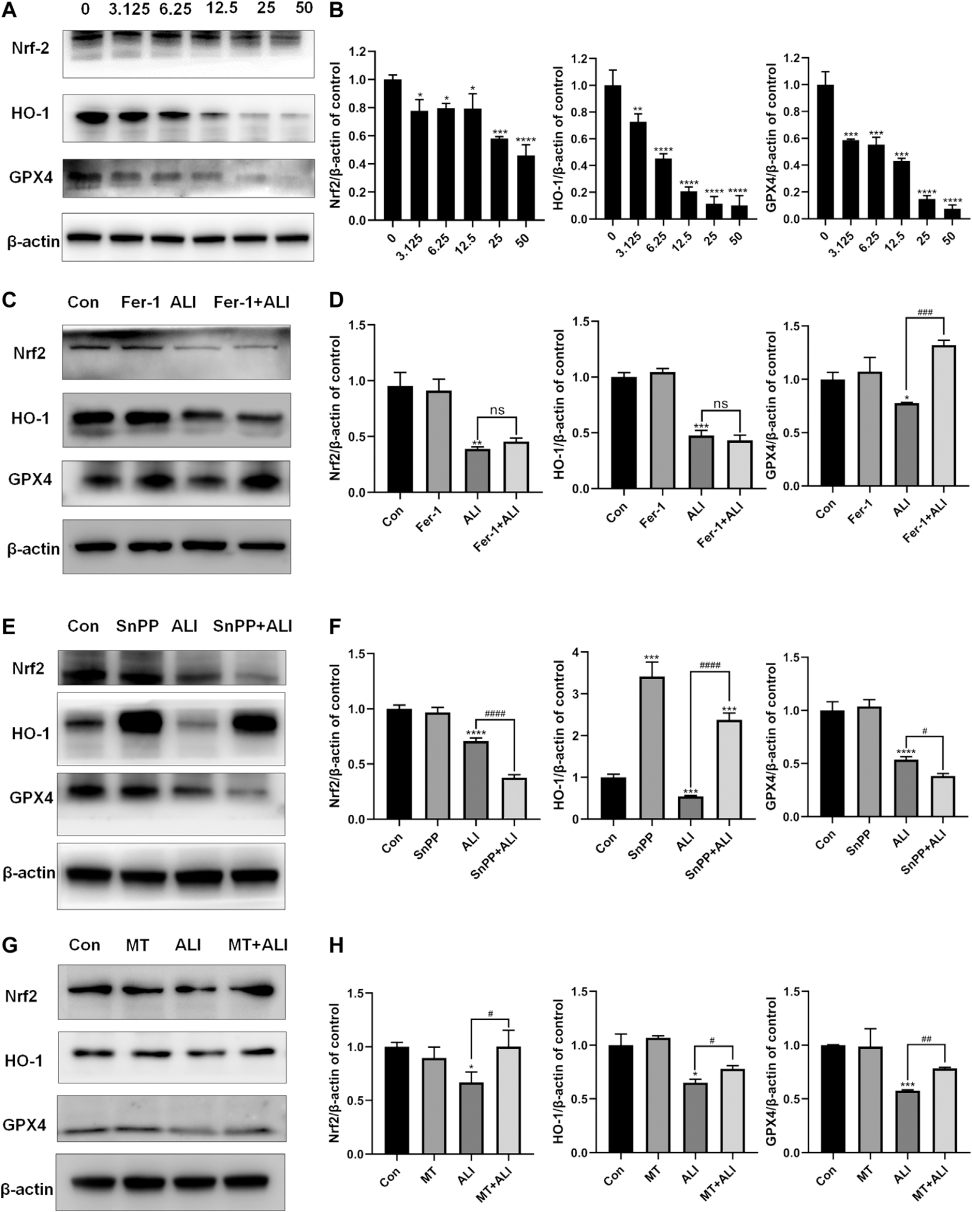
Ferroptosis induced by ALI was related to the inhibition of Nrf2-HO-1/GPX4 pathway. **(A)**, **(B)** The effect of ALI on the protein of Nrf-2, HO-1, and GPX-4 was evaluated by Western blot. **(C)**, **(D)** Western blot was used to detect the effect of ALI on protein Nrf-2, HO-1, and GPX-4 after Ferrostatin-1 pretreatment. **(E)**, **(F)** Protein levels of Nrf-2, HO-1, and GPX-4 in HK-2 cells were detected by Western blot after co-treatment of ALI and SNPP. **(G)**, **(H)** Western blot was used to detect the effects of ALI combined with MT on the protein levels of Nrf-2, HO-1 and GPX-4 in HK-2 cells. ns, no significant; ^*^ *p* < 0.05, ^**^ *p* < 0.01, ^***^ *p* < 0.001, ^****^ *p* < 0.0001 vs. the control group; ^#^ *p* < 0.05, ^##^ *p* < 0.01, ^###^ *p* < 0.001, ^####^ *p* < 0.0001 vs. the ALI group.

To evaluate the role of the antioxidant system in the ALI-induced ferroptosis, Western blot was performed to detect the levels of antioxidant/ferroptosis-related proteins. As shown in Figures 5A and 5B, compared with the control group, the expression of Nrf-2, HO-1, and GPX-4 markedly decreased in the ALI groups in a dose-dependent manner. Fer-1, as a ferroptosis inhibitor by scavenging lipid peroxide, elevated the expression of GPX4 compared with that in the ALI group, but did not affect the levels of Nrf2 and HO-1 (Figures 5C and 5D). SnPP, an HO-1 antagonist, as shown in Figures 5E and 5F, alone could distinctly induce the overexpression of HO-1, and also the co-treatment of ALI and SnPP significantly elevated ALI-downregulated HO-1. But the levels of GPX4 and Nrf2 were both reduced by SnPP co-treatment (Figures 5E and 5F). While as shown in Figures 4B and 4C, SnPP significantly reduced the mitochondrial Fe^2+^ overload induced by ALI. In Figures 5G and 5H, the decreased levels of Nrf2, HO-1, and GPX4 caused by ALI were partly restored in MT and ALI co-treatment group. Therefore, our results suggested that ALI could induce ferroptosis of HK-2 by inhibiting Nrf2-HO-1/GPX4 signaling pathway.

## Discussion

AAN, a kind of renal tubulointerstitial disease associated with aristolochic acid or AAAs, has attracted worldwide attention (Debelle et al., 2008). Over the last 2 decades, although herbal medicines containing aristolochic acid have been banned in many countries, AAN cases remain regularly reported all around the world, and the pathogenic mechanisms involved in AAN are largely unclear (Jadot et al., 2017). ALI, as the major component of AAAs and the principal detoxication metabolite of AAI (Priestap et al., 2012), has been reported to be more nephrotoxic than AAI, but the underlying toxic mechanisms of nephrotoxicity remain poorly understood. In the present study, we firstly showed that ALI could cause ferroptosis in the human proximal tubular epithelial cell, which could be enhanced by induced mitochondrial iron overload via suppressing Nrf2-HO-1/GPX4 mediated anti-lipid hydroperoxidation system, indicating a new toxic mechanism of action of ALI-induced nephrotoxicity.

It has been demonstrated that renal tubular epithelial cell is the main target of AAAs, and AAN is characterized by excessive death of renal tubular epithelial cells (Zhou et al., 2010; Huang et al., 2013). For the past few years, several cell death modes, including apoptosis, necrosis, and autophagy, have been proved to be responsible for AA-induced cell death (Zhou et al., 2010; Baudoux et al., 2012; Zeng et al., 2014; Xie et al., 2017; Yang et al., 2019). While ferroptosis, a new form of regulated cell death, has been reported to be related to various diseases, including kidney injury. However, the role of ferroptosis in AAN is still a virgin ground. Free iron overload, accumulation of lipid peroxides, and mitochondrial morphological characteristics are the three key features that distinguish ferroptosis from other programmed cell death modes (Dixon et al., 2012). In this present study, ALI treatment significantly caused the inhibition of cell viability, being consistent with the previous studies (Li et al., 2010). Moreover, our study showed that the intracellular free iron and 4-HNE, the end-product of lipid peroxidation, both dramatically increased in a dose-dependent manner. Intriguingly, we found that ALI-induced cell death could be more effectively attenuated by DFO and Fer-1, suggesting that compared to apoptosis, necrosis, and autophagy, ferroptosis played a predominant role in ALI-induced renal tubular epithelial death. Apart from increased mitochondrial membrane density, the special morphological changes of ferroptosis, mitochondrial damage characterized by mitochondria swelling, cristae disruption, and vacuolization were also observed in cells treated with ALI, implying ALI-induced excessive oxidative injury of mitochondria accompanying with elevated oxidative stress in mitochondria. We further identified mitochondrial Fe^2+^ overload, which participated in superoxide anion generation in HK-2 cells. As reported, increased mitochondrial labile Fe^2+^ induced excessive ROS possibly through Fenton and Haber–Weiss reaction (Kajarabille and Latunde-Dada, 2019; Nakamura et al., 2019). Taken together, these results revealed that ferroptosis and following mitochondrial iron overload were the principal toxic mechanisms of action in ALI-induced cell death.

Mechanistically, it is generally accepted that direct inhibition of GPX4 and GSH depletion are two key initiators of ferroptosis (Cao and Dixon, 2016). GSH, a simple tripeptide, consists of glutamate, cysteine, and glycine, with the reactive thiol group on cysteine, and GPX4, a glutathione peroxidase, is widely known as an essential anti-ferroptotic mediator (Seibt et al., 2019). Both of them are the most important components of the cellular antioxidant defenses. GPX4 can catalyze GSH to GSSG via the oxidative reaction, during which lipid peroxides are reduced into their less-reactive alcohol form (Yang et al., 2014). In other words, GPX4 could terminate the ferroptotic cascade by using GSH as the substrate. As expected, compared with that in the control group, the expressions of GSH and GPX4 in ALI-treated cells were both dose-dependently declined, supporting that overwhelming ferroptosis-induced lipid peroxidation could not be reversed by cellular GSH antioxidant system.

Aside from GSH and GPX4, the role of the Nrf2-HO-1 signaling pathway is the common inducible antioxidant defense system. The transcription factor Nrf2, a master regulator of the antioxidant system, also plays a critical role in mediating iron/metal metabolism, lipid metabolism, and glutathione synthesis, all of which involve in preventing the initiation of ferroptosis (Dodson et al., 2019; Anandhan et al., 2020). HO-1 is considered as a cytoprotective, anti-inflammatory, and anti-oxidant enzyme, while its role in ferroptosis is debatable (Lever et al., 2016). Of note, GSH, GPX4, and HO-1, all of them could be regulated by the common upstream factor, Nrf2, to exert antioxidant effects (Dodson et al., 2019). Herein, with the dose-dependent decrease of Nrf2 level, the expression of GSH, GPX4, and HO-1 were also significantly reduced, indicating that ALI could inhibit Nrf2-HO-1/GPX4 related antioxidative system. Interestingly, compared with that in the control group, the expression of HO-1 was dramatically increased after co-treatment with ALI and SnPP, but the decreased GPX4 was not alleviated, suggesting that overexpression of HO-1 has a dual effect on ALI-induced ferroptosis. On the one hand, HO-1 could inhibit oxidative damage and mitochondrial iron accumulation induced by ALI. On the other hand, a dramatic increase in HO-1 might also increase iron release from mitochondria or other labile iron pools. Taken together, the inhibition of Nrf2-HO-1/GPX4 axis could be associated with ALI-induced ferroptosis.

Besides, after co-treatment with mitoTEMPO, mitochondrial superoxide anion was decreased, the downregulated expressions of Nrf2, HO-1, and GPX4 were reversed, indicating that ALI caused the inhibition of this cellular antioxidant system mainly by enhancing mitochondrial ROS generation. *De facto,* the kidney is rich in mitochondrial content (Bhargava and Schnellmann, 2017). Excessive mitochondrial ROS-mediated mitochondrial dysfunction is a common and early toxic event in many kidney diseases (Bhargava and Schnellmann, 2017; Galvan et al., 2017). Besides, co-treatment with DFO and ALI obviously inhibited ALI-induced superoxide anion, suggesting that mitochondrial ferrous iron would promote mitochondrial ROS generation. Mitochondria contain iron-containing proteins, such as sulfur-iron clusters, which could be attacked by increased ROS and in turn enhance the free iron release and the Fenton reaction in mitochondria. Hence, our data showed that ALI might cause a vicious cycle of mitochondrial ferrous ion increase and mitochondrial ROS generation, eventually resulted in Nrf2-HO-1/GPX4 antioxidant system inhibition and ferroptosis.

## Conclusion

**FIGURE 6:**
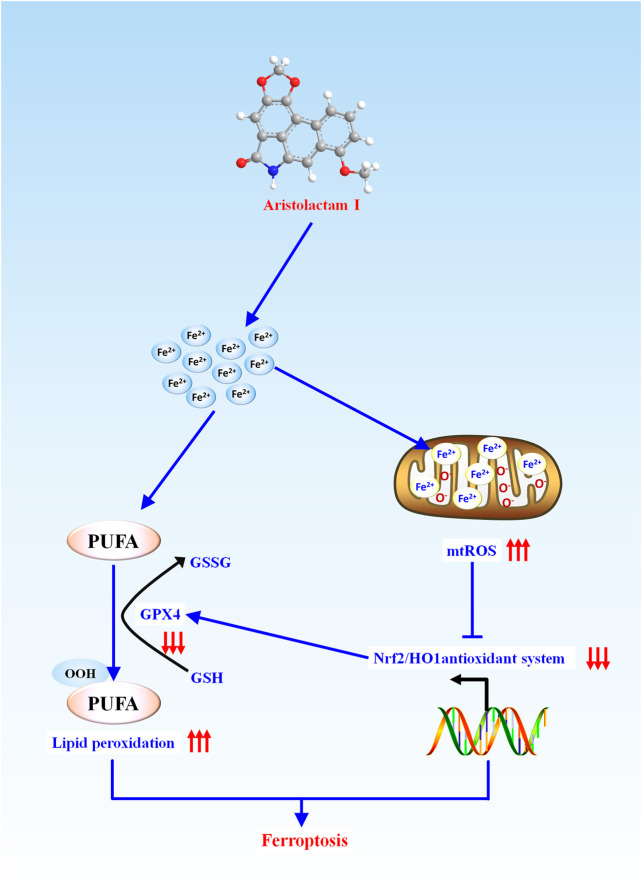
Schematic representation of the potential toxic mechanism involved in ALI-induced nephrotoxicity.

In conclusion, as presented in Figure 6, we firstly demonstrated that ALI caused obvious ferroptosis in HK-2 cells, which was associated with the activation of lipid peroxidation and suppression of the antioxidant system. During this progress, Fe^2+^ overload-mediated mitochondrial ROS over-release would activate lipid peroxidation and inhibit the antioxidant system by inhibiting Nrf2-HO-1/GPX4 pathway, which enhanced ALI-induced ferroptosis. Altogether, our findings provide new insight into toxic mechanisms underlying ALI-triggered nephrotoxicity, and have implications that mitochondrial iron overload mediated-ferroptosis could be an alternative target for the treatment of kidney injury induced by medicine-food homologous varieties containing ALI.

## Data Availability Statement

The original contributions presented in the study are included in the article/Supplementary Material, further inquiries can be directed to the corresponding author.

## Author Contributions

H-FD and Y-HN carried out the experiment. L-XY, C-SH, and L-ZQ used statistical, mathematical or other formal techniques to analyze data. N-NW, Y-QZ, and HL wrote the manuscript. WZ formulated research aims and revised the manuscript. XL revised the manuscript critically for important intellectual content. X-LT, H-LT, Y-GW, and Z-CM designed methodology. YG took the leadership responsibility for the research activity planning and execution. All authors read and approved the final manuscript.

## Funding

This work was supported by grants from the National Key research and Development Program of China (No. 2019YFC1604900).

## Conflict of Interest

The authors declare that the research was conducted in the absence of any commercial or financial relationships that could be construed as a potential conflict of interest.

## Correction note

A correction has been made to this article. Details can be found at: 10.3389/fphar.2025.1734058.

## Abbreviations

AAAs, aristolochic acid analogs; AA, aristolochic acid; AAN, aristolochic acid nephropathy; ALI, aristolactam I; AAI, aristolochic acid I; CCK-8, cell counting kit-8 assay; CKD, chronic kidney disease; DFO, deferoxamine mesylate; Fer-1, Ferrostatin-1; GSH, glutathione; GSSG, glutathione disulfide; GPX4, glutathione peroxidase 4; 4-HNE, 4-hydroxynonenal; HO-1, heme oxygenase-1; 3-MA, 3-Methylamine; MT, mitoTEMPO; mtROS, Mitochondrially generated reactive oxygen species; Nec-1, Necrostatin-1; Nrf2, nuclear factor erythroid 2-related factor 2; PUFA, polyunsaturated fatty acid; ROS, Reactive oxygen species; SnPP, Tin-protoporphyrin IX; TCM, Traditional Chinese medicine
