# Correction: Mitochondrial iron overload-mediated inhibition of Nrf2-HO-1/GPX4 assisted ALI-induced nephrotoxicity

**DOI:** 10.3389/fphar.2025.1734058

**Published:** 2025-12-15

**Authors:** Hui-Fang Deng, Lan-Xin Yue, Ning-Ning Wang, Yong-Qiang Zhou, Wei Zhou, Xian Liu, Yu-Hao Ni, Cong-Shu Huang, Li-Zhen Qiu, Hong Liu, Hong-Ling Tan, Xiang-Lin Tang, Yu-Guang Wang, Zeng-Chun Ma, Yue Gao

**Affiliations:** 1 Department of Pharmaceutical Sciences, Beijing Institute of Radiation Medicine, Beijing, China; 2 Tianjin University of Traditional Chinese Medicine, Tianjin, China; 3 School of Traditional Chinese Medicine, Guangdong Pharmaceutical University, Guangzhou, China

**Keywords:** aristolactam I, nephrotoxicity, ferroptosis, mitochondrial iron overload, Nrf2-HO-1/GPX4

There was a mistake in Figure 2 as published. The image for the DFO group in Figure 2C was mistakenly replaced with the control group’s image, resulting in partial overlap between **Figure 1C** and Figure 2C. Additionally, to prevent misinterpretation of the ALI (25 μM) groups in **Figures 1C** and Figure 2C, we have used new images for the ALI group in Figure 2C. Finally, the housekeeping protein β-actin in Figure 2E was inadvertently duplicated from **Figure 5C**, and we have corrected this error. The corrected Figure 2 appears below.

**FIGURE 2:**
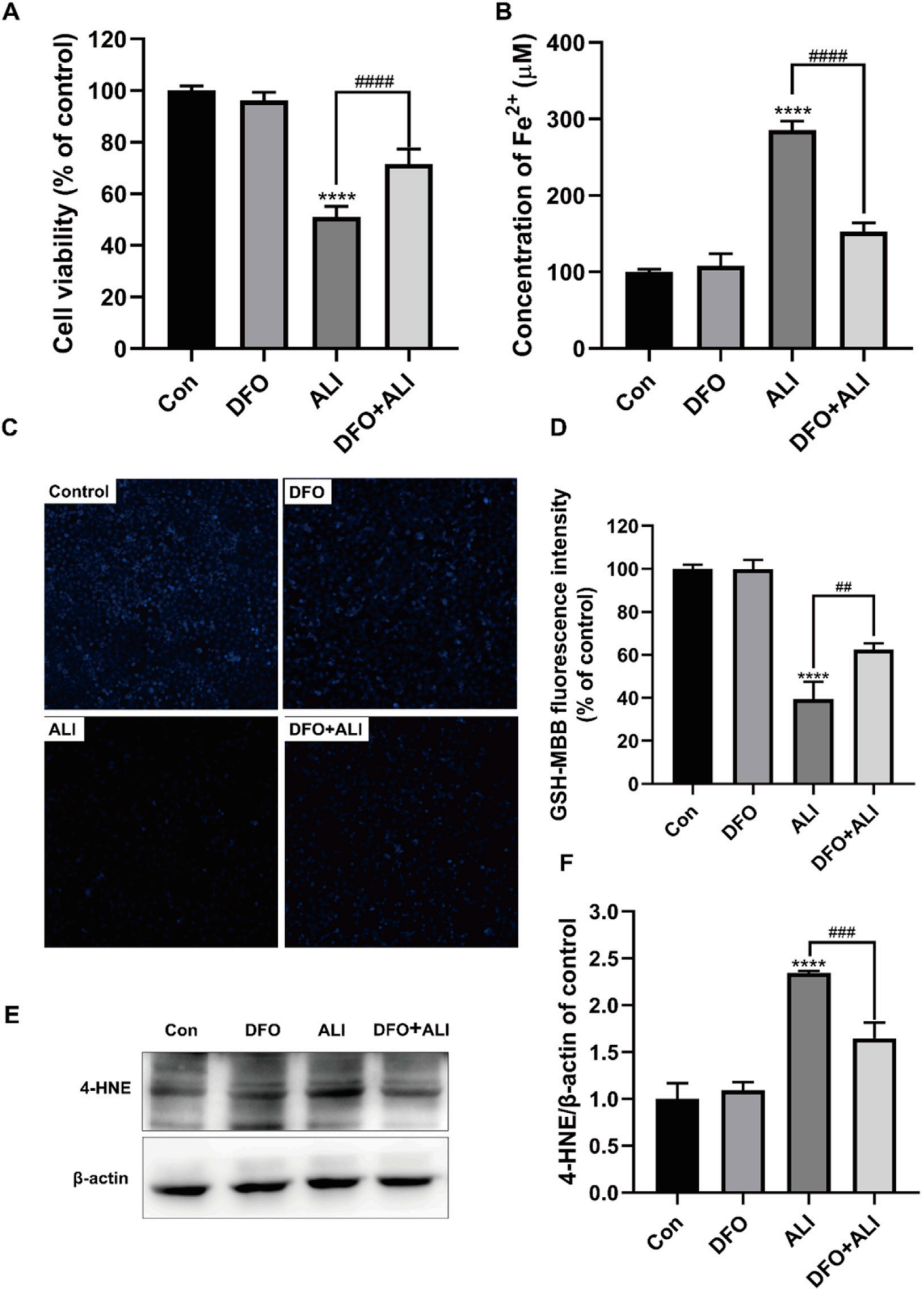
ALI-induced cytotoxicity could be alleviated by iron chelator DFO. **(A)** Cell viability of HK-2 cells was detected using CCK-8 assay. **(B)** Intracellular Fe^2+^ levels in HK-2 cells treated by ALI and DFO. **(C,D)** Intracellular GSH content in HK-2 (X200). **(E,F)** The protein levels of 4-HNE were measured by Western blot. ^*^ *p* < 0.05, ^**^ *p* < 0.01, ^***^ *p* < 0.001, ^****^ *p* < 0.0001 vs. the control group; ^#^ *p* < 0.05, ^##^ *p* < 0.01, ^###^ *p* < 0.001, ^####^ *p* < 0.0001 vs. the ALI group.

The original article has been updated.

